# Caring for adults with CHD in the era of coronavirus disease 2019 pandemic: early experience in an Italian tertiary centre

**DOI:** 10.1017/S1047951120002085

**Published:** 2020-07-06

**Authors:** Giancarlo Scognamiglio, Flavia Fusco, Assunta Merola, Michela Palma, Anna Correra, Berardo Sarubbi

**Affiliations:** Adult Congenital Heart Disease Unit, Department of Cardiology, Monaldi Hospital, Naples, Italy

**Keywords:** Adult Congenital Heart Disease, coronavirus disease 2019, pandemic, healthcare, hospital admission

## Abstract

**Background:**

Adults with CHD are known to greatly benefit from a prompt access to continuous expert care. On the other hand, coronavirus disease 2019 pandemic has determined a dramatic worldwide reconfiguration of the healthcare systems, with rapid redeployment of resources towards this emergency. Italy was the first Western country affected by a large-scale spread of coronavirus disease 2019. The aim of our study is to analyse the impact of the coronavirus disease 2019 outbreak on in-hospital care of patients with CHD in an Italian tertiary centre.

**Methods and results:**

We retrospectively reviewed data on CHD hospital admissions in our centre since 1 March, 2020, when the adoption of a strict infection containment policy throughout the country resulted in limited access of patients to routine hospital care and resources reallocation to the care of infected patients. Comparison with data from the previous year was performed in order to identify any relevant differences attributable to the outbreak. Despite cancellation of all elective procedures, the overall number of urgent hospital admission remained stable throughout the period of study. Patients admitted during the pandemic had greater disease complexity (p = 0.001) with longer length of in-hospital stay (p = 0.01). No adverse events or positive swabs were reported among CHD patients who were admitted to hospital or medical personnel caring for these patients.

**Conclusion:**

Data from our early experience suggest that coronavirus disease 2019 pandemic did not impact significantly on the provision of urgent care to adult patients with CHD.

The recent outbreak of coronavirus disease 2019 has rapidly determined a worldwide crisis of the healthcare systems. Italy was the first Western country to be affected by a large-scale spread of coronavirus disease 2019. Italian Government enforced a nationwide lockdown since 9 March, 2020 in order to flatten the curve. At the same time, hospitals throughout the country were suddenly challenged to restructure the model of care, adopting emergency protocols to contain the exposure while also providing for an increasing number of patients with severe forms of coronavirus disease 2019 infection.

Despite the dramatic improvement in life expectancy achieved in the last decades, adults with CHD may have residual cardiac lesions and require lifelong expert care. Moreover, these patients may have complex cardiac physiology and may easily deteriorate in case of any intercurrent disease, which could disrupt their fragile haemodynamic and trigger arrhythmic events.

From early reports, patients with cardiovascular disease appear to have higher mortality and morbidity rate in case of coronavirus disease infection,^1^ with emerging evidence of possible cardiac involvement.^2,3^ However, specific data on the impact of the coronavirus disease 2019 outbreak on the system of care of patients with CHD and their outcomes are still lacking. Accordingly, we reviewed the data on adults with CHD admitted to our tertiary centre before and during the pandemic in order to describe our early results in adjusting the healthcare model to this new scenario.

## Materials and methods

### Patients inclusion

Electronic CHD database was retrospectively searched for all consecutive patients admitted to our institution during the coronavirus disease 2019 pandemic. The study period was defined as the time between the date of adoption of the infection containment protocol in our institution (1 March, 2020) and 30 April, 2020. Patients admitted during the same period of the previous year were included for comparison, in order to identify any relevant changes attributable to the outbreak in terms of underlying disease complexity, urgency, main reason and modality of hospital admission, and length of in-hospital stay. Patients aged <18 years were excluded. Disease complexity was assessed according to the classification of Task Force 1 of the 32nd Bethesda conference.^4^

### In-hospital infection containment protocol during the coronavirus disease 2019 pandemic

Since 1 March, our institution started to admit patients with coronavirus disease within dedicated departments and pathways, separated from non-coronavirus disease patients. At the same time, all the previously scheduled elective procedures were cancelled, in accordance to the Government indications on the in-hospital care during the coronavirus disease 2019 emergency.^5^ Therefore, only patients deemed in need of urgent care after review by the CHD multidisciplinary team were admitted. In case of emergency, patients were instructed to preferably seek medical advices with the use of technological tools including phone calls, e-mail, and video conference, when feasible, in order to reduce non-essential attendance at emergency department and possible viral exposure. Furthermore, in agreement with the national guidelines on coronavirus disease 2019 prevention in health facilities,^6^ all patients on admission were screened for symptoms suggestive of coronavirus disease 2019 and underwent nasopharyngeal swab. Patients admitted were isolated in a single room pending the results of analyses. Other infection control measures included: universal testing, visiting restriction, patients and families education, use of personal protective equipment, special hygiene procedures, staff training, and focused clinical and echocardiographic exams.

### Statistical analysis

Statistical analysis was performed using Medcalc version 19.2.0 (MedCalc Software, Ostend, Belgium). Continuous variables were reported as mean ± standard deviation or median (IQR), according to data distribution. Data normality was assessed with Shapiro–Wilk test. Pre- and post-outbreak differences were assessed by paired Student t-test or Mann–Whitney signed-rank test, as appropriate. Categorical variables including disease complexity, reason for hospital admission, priority level, and modality of admission were reported as frequencies (%) and were compared by χ^2^ test. P-values <0.05 were considered statistically significant.

## Results

### Hospital admission before the pandemic

**Figure 1.**
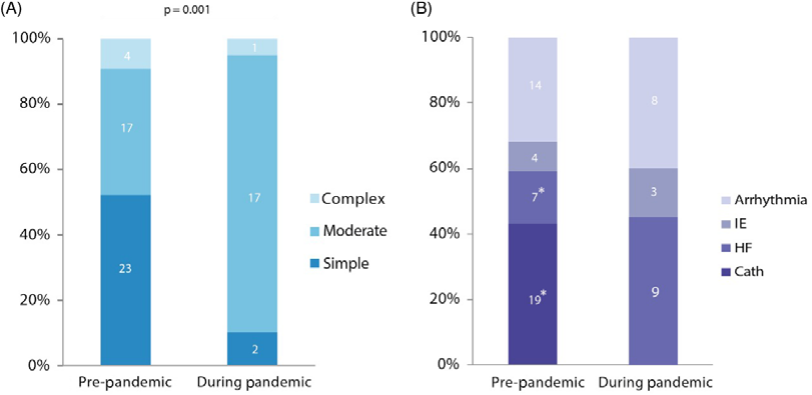
Percentage changes in reason for hospitalization and disease complexity of the patients admitted in our CHD tertiary centre during the Coronavirus disease 2019 pandemic (March-April 2020) and in a corresponding period of the previous year. (A) Disease complexity according to the Task Force 1 of the 32nd Bethesda conference (4) of the adult patients with CHD admitted to our institution during the period of study. Patients admitted during the pandemic had significantly greater disease complexity compared to the pre-pandemic period (simple vs moderate/complex defect p = 0.001). (B) Primary reason for hospital admission of adult patients with CHD. Asterisks indicate p-values<0.05 between subgroups. Cath = Catheterization; HF = Heart failure; IE = Infective endocarditis.

Data on patients admitted to hospital in the pre-pandemic period are reported in Table 1 and Figure 1. Elective hospitalisations accounted for the vast majority (70%) of hospital admissions. Patients were mainly admitted to undergo elective procedures, which were scheduled after face-to-face visits in the outpatient CHD clinic and multidisciplinary team review. Other modalities of admissions included referral from other hospitals and urgent admissions from the emergency department or the outpatient clinic.

**Table 1.** Hospital admissions in our tertiary CHD centre during coronavirus disease 2019 pandemic (March–April 2020) and during the same period of the previous year

	March–April 2019 (N = 44)	March–April 2020 (N = 20)	p-value
Age	46.3 ± 17.3	48.4 ± 15.4	0.7
Sex	26 (59%) male	12 (60%) male	0.9
Priority level	13 (30%) urgent 31 (70%) elective	20 (100%) urgent	<0.001
Modality of admission	29 (65%) scheduled 10 (23%) emergency department 2 (5%) external referral 3 (7%) adult CHD outpatient clinic	0 scheduled 15 (75%) phone contact 3 (15%) external referral 2 (10%) cardiac implantable electric devices outpatient clinic	<0.001
Length of in-hospital stay	3 (2.7–4.3)	4 (3–10)	0.01

### Impact of the coronavirus disease 2019 pandemic on hospital admissions of CHD patients

Demographics and baseline characteristics of our population as well as details on hospital admission before and during coronavirus disease 2019 outbreak are summarised in Table 1 and Figure 1. The two groups did not differ significantly in terms of age (p = 0.7) and sex distribution (p = 0.9).

The global number of hospital admissions during the coronavirus disease 2019 pandemic was reduced by 55% compared to the same period of the previous year (20 versus 44). However, the number of patients requiring urgent medical attention remained stable. Notably, a significant increase in the level of complexity of the underlying CHD was reported among the patients admitted during the pandemic compared to the same months in 2019 (simple versus moderate/complex defect p = 0.001, Fig 1 A). In addition, the infection containment protocol resulted in a substantial change in the modality of admission: during the outbreak, the indication to hospitalisation was given in 75% of cases after phone/video call with our staff, followed by urgent patient assessment in the outpatient clinic by a CHD-specialised cardiologist to confirm the necessity of hospitalisation. The remaining patients were admitted from the outpatient clinic in two cases and after external referral in three cases. During the pandemic, decompensated heart failure was the primary reason for admission in the vast majority of patients (nine patients, 45%). Eleven (55%) patients required an invasive procedure: three pacemaker battery replacement, one pacemaker implantation, one loop recorder implantation, four atrial septal defect closure, one percutaneous closure of ruptured sinus of Valsalva, one diagnostic catheterisation. The indication to urgent atrial septal defect closure was paradoxical embolism in one patient, refractory supraventricular arrhythmias in one patient, and decompensated heart failure in two patients. Although the length of in-hospital stay was longer during the coronavirus disease 2019 pandemic (p = 0.01), no major adverse events or death occurred among the patients hospitalised throughout the two periods in exam. Importantly, no positive swabs were reported among admitted CHD patients or hospital staff caring for these patients.

## Discussion

Our single-centre early experience in managing adults with CHD during the coronavirus disease 2019 pandemic suggests that

- despite the restriction of non-critical admissions, delivery of care for urgent issues can be successfully guaranteed in specialised tertiary centres with the adoption of a stringent infection containment protocol;
- in spite of prominent concerns about viral transmission, adults with CHD, especially those with complex disease, continue to seek expert medical attention, as opposite to patients with acquired heart disease;
- continuity of non-urgent expert care to patients with CHD during the pandemic was mostly ensured thanks to the use of technology-based alternative assessment modalities.

Based on the evidence of more severe coronavirus disease 2019 impact on patients with acquired heart disease, it is reasonable to assume that CHD patients might be particularly vulnerable and potentially seriously compromised by this infectious disease. Therefore, prevention of viral transmission is crucial in this population.^7^ These patients may also present additional risk factors such as propensity to thrombotic events, susceptibility to heart failure and arrhythmias, depressed immunological status, and polipharmacy, which could complicate the disease course in case of infection.

The absence of patients tested positive in our series confirms the importance and efficacy of both strong hospital commitment to an infection containment policy and patients’ education on the measures to minimise the risk of transmission.

Globally, our data on the hospital admissions, disease severity, and length of in-hospital stay during the pandemic provide further evidence of the constant need of expert care for adults with CHD in specialised centres. Limitation of in-person contact and necessity of resources reallocation to coronavirus disease 2019 patient forced us to postpone all the scheduled elective procedure. Analogous restriction of the invasive procedures was recently reported by a survey on cardiac catheterisation in patients with CHD during the coronavirus disease 2019 pandemic in the United States of America.^8^ The authors concluded that scaling back non-critical catheterisation and performing only procedures deemed urgent might be reasonable in this worldwide healthcare crisis. In our early experience during coronavirus disease 2019 pandemic, the cancellation of the elective procedures did not impact significantly on delivery of urgent care to patients with CHD.

The emergency departments have become the epidemic’s frontline all over the world. Consequently, patients with non-urgent and non-coronavirus disease 2019-related issues were advised against attending the emergency departments, overwhelmed by patients presenting with suspected coronavirus disease 2019 infection. Therefore, in our Adult Congenital Heart Disease Unit, we are currently responding to the patient requests of medical attention embracing new alternative ways of communication, including phone/video consultations and using data collected from remote monitoring devices. These modalities allowed us to assess the general health status, to collect essential data on vital signs including heart rate and rhythm, blood pressure and oxygen saturation and to discuss the symptoms and adherence to medications for routine evaluation of patients with stable conditions and to make individualised decisions about the priority level for hospital admission for unstable patients.

A further emerging concern is that patients with acute cardiac conditions might be reluctant to visit hospitals, being scared of potential viral exposure. This is an extremely dangerous behaviour, which may lead to significant diagnostic delay and subsequent severe complications. This trend has already been largely reported in patients with acute coronary syndromes,^9,10^ with the evidence of a substantial drop of hospitalisations during the pandemic. Nevertheless, in our series, the fear of this potentially fatal infection did not affect the number of urgent hospital admissions in our tertiary centre and patients with CHD and acute complications such as decompensated heart failure and arrhythmias kept seeking medical attention with the use of alternative modalities. Again, health-related education provided by CHD-specialised centres may account for these differences with patients with acquired heart disease. CHD patients, educated since childhood to recognise subtle changes in their status, may develop a higher level of awareness of their cardiac conditions, which enables them to promptly identify red-flag symptoms and seek medical attention earlier. However, only long-term multicentre studies will reveal whether a restriction of non-urgent hospital admissions will result in an increased mortality rate among coronavirus disease-negative CHD patients.

The current challenges of managing complex patients in the era of coronavirus disease 2019 outbreak offer us the opportunity to redesign our health services and develop long-term strategies to deal with infection containment, while continuing to ensure high-quality care for our patients. Health model restructuring should go through a reasoned resources reallocation,^11^ promotion of international collaborations, and use of new technologies such as remote monitoring, remote counselling, and artificial intelligence to allow early detection of disease-modifying events.^12^ Moreover, further investment on patient education to social distancing is paramount,^13,14^ particularly moving forward to the next stage of the pandemic, during which more patients with CHD will inevitably be exposed to coronavirus disease 2019.

## Limits

In our population, the number of patients requiring urgent medical attention remained stable during coronavirus disease 2019 outbreak. However, our results may be biased by the limited number of patients, the short period of observation, and the short period between the initial alterations of our clinical activities due to coronavirus disease 2019 and data collection. Further studies with larger cohort and longer follow-up are needed to confirm our findings and to investigate whether the current changes in our clinical activities will result in an increased out-of-hospital mortality among adults with CHD.

## Conclusions

Our early experience demonstrated that the adoption of a stringent infection containment protocol, along with patient education, allows safe management of adults with CHD admitted for urgent care in specialised tertiary centres during the coronavirus disease 2019 pandemic. Furthermore, in our series, unlike patients with acquired heart disease, patients with CHD kept seeking medical attention appropriately in case of need, by means of novel technological modalities to contact the CHD team. Therefore, development of novel management pathways and strategies is mandatory to contain viral in-hospital transmission and ensure uninterrupted integrated care to this complex population through the next steps of the pandemic response.
